# Effect of a Bacterial Laccase on the Quality and Micro-Structure of Whole Wheat Bread

**DOI:** 10.4014/jmb.2305.05008

**Published:** 2023-07-28

**Authors:** Jingjing Wang, Han Bai, Ran Zhang, Guoao Ding, Xuran Cai, Wei Wang, Guilan Zhu, Peng Zhou, Yan Zhang

**Affiliations:** 1 School of Life Sciences, Hefei Normal University, Lianhua Road 1688, Hefei 230601, Anhui, P.R. China; 2 Department of Life Science, Anhui University, Hefei 230061, P.R. China

**Keywords:** Whole-wheat bread, laccase, gluten-free protein, cross-linking

## Abstract

The gluten protein content in whole-wheat flour is low, which affects the elasticity and viscosity of the dough. Enzymatic modification of the protein may result in a network that mimics gluten, which plays an important role in the processing of whole-wheat foods. In this study, the effects of *Halomonas alkaliantartica* laccase (LacHa) on the quality parameters of whole-wheat bread were investigated. The optimum dosage of LacHa was 4 U/100 g of whole-wheat flour. At this dosage, whole-wheat bread exhibited the best specific volume and optimum texture parameters. Laccase also extended the storage duration of whole-wheat bread. We analyzed the micro-structure of the dough to determine its gluten-free protein extractable rate and free sulfhydryl group content, and verify that LacHa mediates cross-linking of gluten-free proteins. The results demonstrated that the cross-linking of gluten-free protein by LacHa improves the texture of whole-wheat bread. As a flour improver, LacHa has great developmental and application potential in baked-food production.

## Introduction

The morbidity of obesity, cardiovascular disease, and diet-related diseases is increasing at an alarming rate worldwide [1-3]. Carbohydrate-rich processed foods containing cooked starch, such as wheat flour noodles, have been shown to cause a rapid increase in postprandial glycemic response, which remarkably increases the risk of diet-related disease over time [4, 5]. In recent years, many studies have shown that replacing wheat flour with whole-grain flour can reduce the glycemic index in the middle of the day and the risk of type 2 diabetes [6, 7].

Whole-wheat flour is a type of flour ground from the entire wheat kernel, without removing the bran, and is a natural source of water-soluble dietary fiber, which can lower cholesterol and control blood sugar levels. It is therefore considered “a special flour type for diabetics” [8, 9]. Whole-wheat flour is fat-free, low in calories, rich in complex carbohydrates, and contains high amounts of vitamins B and E, potassium, selenium, and iron, all of which can help prevent obesity [10]. Moreover, the addition of whole wheat to food not only contributes to meeting the nutritional needs of consumers but also provides a new way to develop functional staple foods [11, 12]. Although whole-wheat flour has high nutritional value, it is not conducive to storage and the taste is relatively poor. The gluten protein content in whole-wheat flour is low, which affects the elasticity and viscosity of dough and plays an important role in the processing of whole-wheat food [10-12]. Adding exogenous proteins to foods such as black barley and sorghum can improve the cross-linking of proteins and improve their quality [13]. Furthermore, the application of enzymatic cross-linking in food processing is widely regarded as a green process technology [14]. Based on previous reports, transglutaminase can strengthen the gluten protein network and improve the mechanical properties of Chinese noodles [15]. Meanwhile, tyrosinase and laccase have been used to modify gluten-free oat dough and gluten-free flour [16]. Among them, laccase has greater potential because of its wider substrate range [13, 17, 18].

Laccase is a multicopper oxidase that catalyzes the one-electron oxidation of a variety of substrates. It is considered an environmentally friendly enzyme [19]. Since water is the only product of the laccase reaction, there is little impact on the environment, and thus laccase is widely used in the food industry [16]. For example, the taste and flavor of baked goods depend on the disulfide bonds in gluten proteins [16, 20]. Laccase oxidizes sulfhydryl groups into disulfide bonds during dough baking. This increases the number of disulfide bonds, thereby strengthening the gluten structure in wheat dough and improving the functional quality, volume, and softness of baked products [21, 22]. The processability of wheat dough can also be improved by increasing its strength and stability and reducing adhesiveness [23, 24]. Whole-wheat flour is rich in phenolic compounds [15, 25], which are potential substrates for laccases that improve the gluten network in whole-wheat foods. The content of thiols and total phenols in dough decreases by 28% and 93%, respectively, with an increase in laccase activity [23, 26-29]. Laccase catalyzes the cross-linking of protein and phenolic-esterified polysaccharides, which improve the gluten network structure and optimize the elastic properties of dough. At present, laccase-induced protein cross-linking is primarily used to discuss the impact on processing characteristics and quality [16, 21, 23].

Currently, most of the laccases applied in food baking come from fungi [15-26]. In this study, we used *Halomonas alkaliantartica* laccase (LacHa), derived from deep-sea bacteria, to prepare whole-wheat bread and explore the effect of the enzyme on bread quality. We also determined the specific volume, texture parameters, and storage properties of bread, as well as the total gluten protein extractable rate and the free sulfhydryl group content of the dough. Combined with the scanning electron microscopy (SEM) results, the findings showed that LacHa as a flour improver has a remarkable impact on the properties of whole-wheat bread.

## Materials and Methods

### Materials

*H. alkaliantartica* laccase (LacHa) was isolated and expressed in *Escherichia coli* in a previous study and the manufacturer’s specification of its activity was 258.6 U/L. LacHa activity was assayed according to the method described by Fang [30]. One unit of enzyme activity was defined as the amount of enzyme required to oxidize Syringaldazine (SGZ) at a rate of 1 μM/min. Protein concentration was determined with the Bradford Protein Assay Kit (TaKaRa, China) with bovine serum albumin as the standard. Whole-wheat flour was manufactured by Xinliang Grain and Oil Processing Co., Ltd. (China) and contained 20% starch, 21% protein, 4% fat, and 49%dietary fiber. Yeast was manufactured by Angel Yeast Co., Ltd. (China). Corn oil was manufactured by Qinglong Gaoke Co., Ltd. (China).

### Dough Preparation

As experimental groups, breads were made of 100 g of whole-wheat flour, 1 g of yeast, and 2 g of vegetable oil with different amounts of LacHa reaction mixture added (1 ml of enzymatic reaction mixture was composed of 20 μl of appropriately diluted enzyme stock and 980 μl of 50 mM pH 7.5 Na_2_HPO_4_-KH_2_PO_4_ buffer). Two randomly selected bread groups were set up as control groups. One was not supplemented with LacHa or Na_2_HPO_4_-KH_2_PO_4_ buffer, while Na_2_HPO_4_-KH_2_PO_4_ buffer was added to the other. These were labeled as C1 and C2, respectively. Based on previous experimental data, the amount of LacHa added in the experimental groups was 1 U, 2 U, 3 U, 4 U, and 5 U. All the ingredients were mixed well, and the doughs were mixed with a bread maker (Petrus, China) at 25°C for 4 min at slow speed (70 r/min).

### Microscopic Examination of the Doughs

Following the method of Bonet *et al*. [31], pieces of the doughs were broken off for testing after freeze drying; the internal powder of the samples to be tested was scraped off, and then fixed on the stage using conductive tape. The powder that was not completely fixed was blown off with an ear-washing ball, and two rounds of gold-plating treatment were carried out on the surface of the samples to be tested for approximately 6 min by using an ion sputtering instrument. The stage was removed and placed in a Zeiss scanning electron microscope chamber for vacuumizing and pressurizing (Hitachi Co., Ltd., Japan). Cross-sections of the samples were observed at 500 × and 1,000× magnification.

### Extractability Determination of Gluten Protein

The extractable rate of gluten protein was determined by size exclusion high-performance liquid chromatography (SE-HPLC; Agilent, USA), and the degree of gluten protein cross-linking was determined in accordance with the test method proposed by Wagner *et al*. [32]. Briefly, the freeze-dried bread samples were dissolved into 1.0 ml of sodium phosphate buffer (0.05 M, pH 7.0), containing 1.0% (w/v) sodium dodecyl sulfate (SDS) with continuous oscillation for 1 h. For reducing condition, 1.0% (w/v) dithiothreitol (DTT) was added into the sodium phosphate buffer. The supernatant was collected and filtered through a 0.45 μm filter membrane for later use. The 300-5C8 chromatographic column (Nucleosil, Germany) was used. The mobile phase was ultrapure water containing 0.1%trifluoroacetic acid (solution A) and acetonitrile containing 0.1% trifluoroacetic acid (solution B). The elution condition was binary high-pressure gradient elution. The concentration of solution B in the eluent was 24.0% to 56.0%. The total flow rate of the mobile phase was 1 ml/min; the column temperature was 50°C; the injection volume was 100 μl, and the UV detection wavelength was 214 nm. The extractability content of proteins in SDS buffer of baked bread was calculated as the ratio of the peak area of the experimental sample compared with the peak area of the control sample. The control sample was extracted under reducing conditions.

### Free Sulfhydryl Group (SH) Determination of the Dough

Freeze-dried dough samples were dissolved into 10 ml 0.2 mol/l Tris buffer (pH 7.5), and extracted under oscillation for 1 h at room temperature. After centrifugation at 15,000 ×*g* for 10 min, 0.1 ml of 10 mmol/l 5, 5-dithiobis (2-nitrobenzoic acid) (DTNB) was added into the suspension (5.0 ml) and reacted for 25 min at room temperature. The values of yellow products were determined at 412 nm with a UV–Vis spectrophotometer (UV-5200, Shanghai Yuanxi Instrument Co., Ltd., China). The content was calculated using a calibration curve made with reduced glutathione, and the control sample was without the DTNB. The free SH was determined according to the method described by Beveridge, Toma, and Nakai [33].

### Baking Procedures

The effects of enzymes on the whole wheat breads were studied by baking breads with a straight dough baking method. All dry ingredients were mixed together and placed in a bread maker (Petrus, China). The yeast was suspended in water (26°C) and added to the dry ingredients. The dough was mixed at low speed (100 r/min) for 2 min, and then at fast speed (200 r/min) for 5 min. After intermediate proofing in an incubator for 50 min (20°C, 80% humidity), the doughs were exhausted, divided, shaped, placed into a mold, attached to a layer of preservative film, and proofed again for 1 h (37°C, 85% humidity). The proofed doughs were baked in a high-temperature oven (160°C on the top and 180°C on the bottom) for 18 min. The bread samples were then cooled to room temperature before being placed into packaging bags.

### Determination of the Specific Volume of Breads

The specific volume of the breads was determined by millet exclusion. A large beaker was filled with millet, and the volume of the beaker was recorded as V1. The weight of the baked and cooled bread was recorded as m. Most of the millet was then poured out of the beaker, leaving a little millet at the bottom (to approximately 3 cm). Next, the sample to be tested was placed into the beaker, which was then filled to the brim again with the millet (being careful not to leave a gap). The volume of the remaining millet was recorded as V2. The specific volume ρ (ml/g) of the bread was determined in accordance with the following formula:

$$\text{ρ=}\frac{{\text{(V}}_{\text{1}}{\text{-V}}_{\text{2}}\text{)}}{\text{m}}$$

### Texture Analysis of the Breads

Texture analysis of the whole wheat bread in the experimental and blank control groups was conducted to accurately determine the impact of LacHa on bread quality using XT. A plus texture analyzer (Chaoji Instrument Co., Ltd., China) was used to analyze the texture characteristics of the bread to determine texture parameters, including hardness, chewiness, cohesiveness, elasticity, and resilience. The data are objective, and they have an important reference value. The baked bread was cooled, and the bread samples were simply shaped to remove the crust on their surfaces. Considering that the crust was too hard to affect the experimental results, the bread was cut into samples of 50 mm in length, 30 mm in width, and 30 mm in height using a bread knife. The processed bread was tested on the test platform of the texture analyzer using the probe P/36R under the following conditions: speed before test of 1.0 mm/s, test speed of 2.0 mm/s, trigger point load of 5.0 g, row variable of 50%, and interval of 30 s. Then, the bread was cycled two times for texture analysis. Each sample was measured three times, and the average value was analyzed according to the method described by Chen (2019).

### Storage Analysis of the Breads

The baked whole wheat bread was naturally cooled and the bread slices (length: 50 mm, width: 30 mm, height: 30 mm) were used for the storage analysis. The bread samples were placed into packaging bags for storage. The hardness, elasticity, resilience, adhesiveness, chewiness and other data of the whole-wheat bread were measured every 24 h by using a texture analyzer, and the results were averaged for 3 consecutive days (the general shelf life of bread without any ingredients is 3 days).

### Data Analysis and Statistics

Statistical significance was assessed using one-way ANOVA by IBM SPSS Statistics 23, and differences between means were determined by Tukey's multiple comparison post hoc analysis (*: *p* < 0.05; **: *p* < 0.01).

## Results

### Microscopic Examination of the Doughs

**Fig. 1:**
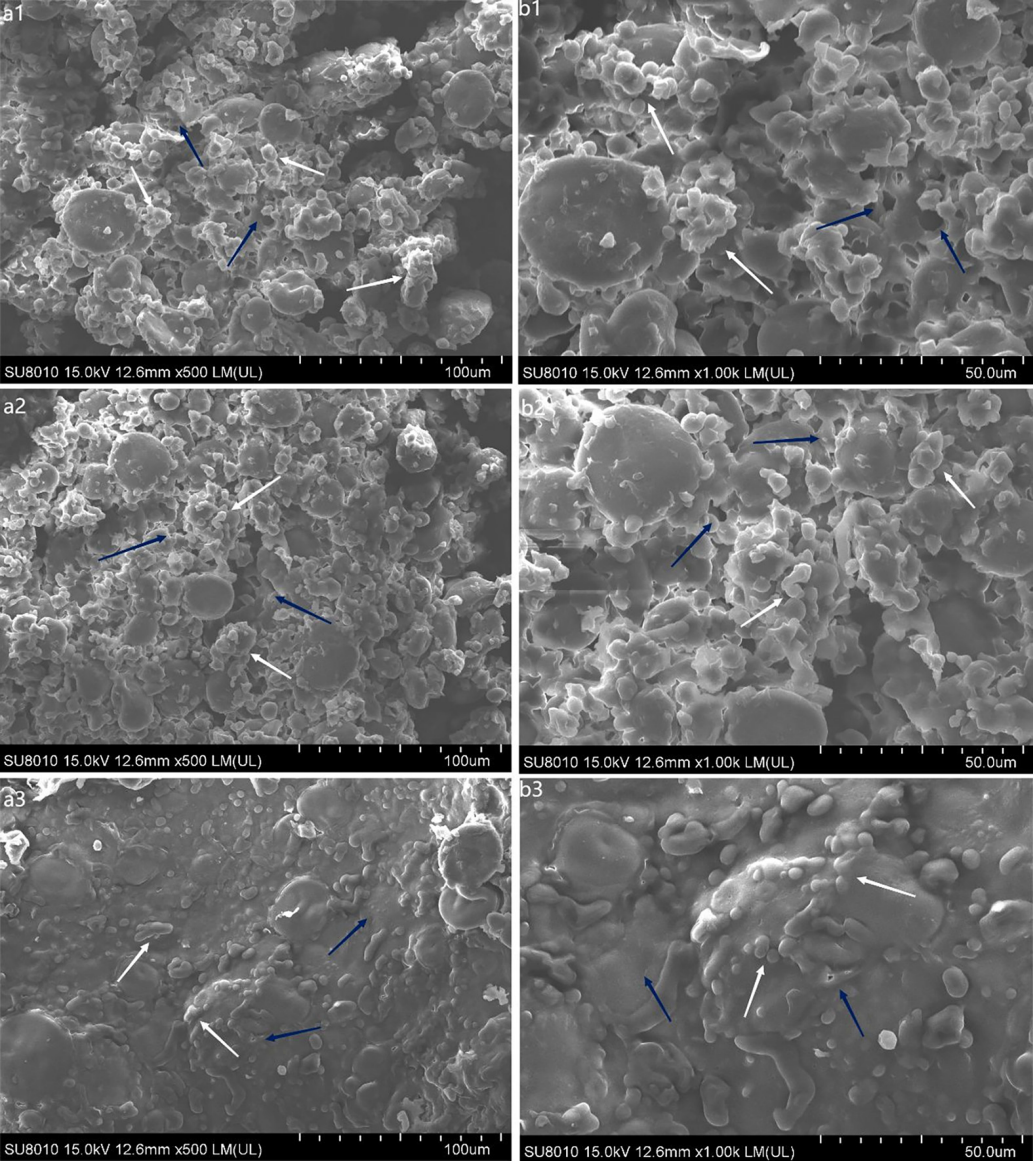
SEM images of whole-wheat dough with different additions of LacHa at 500× (a) and 1,000× (b) magnification. The subscript 1-3 represents the dough with whole wheat-added sterile water, whole wheat added-phosphate buffer, and whole wheat-added 4 U LacHa. The areas highlighted with white arrow and blue arrow show the exposed starch granules and large cracks, respectively.

The scanning electron microscope (SEM) is a popular optical tool that can intuitively determine the changes in dough microstructure. A rough surface, exposed starch granules (highlighted with white arrow), and large cracks (highlighted with blue arrow) were observed in the blank control sample and in the control group with the addition of phosphate buffer (Fig. 1). Meanwhile, numerous starch granules were crushed and scattered through the rough surface. Conversely, whole-wheat dough acquired a smoother and denser structure after LacHa addition (Figs. 1a and 1b). In the experimental group, starch granules were more embedded in the whole-wheat dough (highlighted with white arrow), whereas crevices and holes became less embedded (highlighted with blue arrow). Therefore, LacHa addition could tightly combine gluten and starch granules, leading to the formation of a smooth, flat structure.

### Analysis of the Extractable Rate of Gluten Protein

**Fig. 2:**
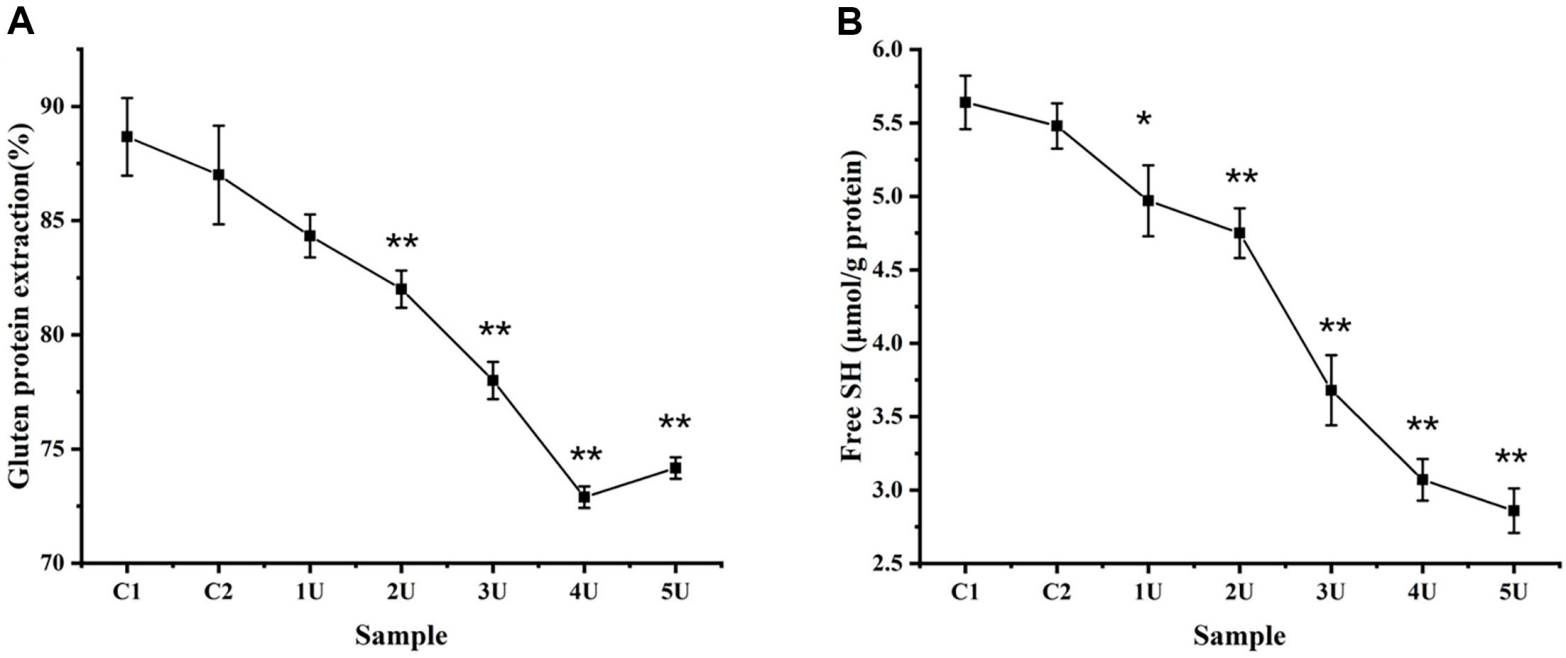
Extraction rate of gluten protein (A) and free sulfhydryl group content of the doughs (B). C1 control group represents the whole wheat-added sterile water, and C2 control group represents the whole wheat added-phosphate buffer. The 1 U, 2 U, 3 U, 4 U, and 5 U represent the experimental groups with laccase addition of 1 U, 2 U, 3 U, 4 U, and 5 U in each group, respectively (*p < 0.05, **p < 0.01, one-way ANOVA, *n* = 3).

The extractable rate of total gluten decreases with an increase in the amount of LacHa added. The extraction rate of all the experimental groups was significantly lower than that of the control group when the dosage of laccase was greater than 1 U, and the data reached a minimum when 4 U LacHa was added (Fig. 2A). Compared with the control group, the extraction rate of whole wheat bread gluten protein with 4 U LacHa decreased from 88.67% to 72.8% (Fig. 2A). However, when the amount of LacHa increased to 5 U, the extraction rate of gluten protein increased slightly, reaching 74.1%.

### Determination of the Free Sulfhydryl Group Content of the Dough

The effects of LacHa on whole-wheat dough were also studied by quantifying the free sulfhydryl group content. We observed that the free sulfhydryl content of the doughs decreased significantly with the addition of LacHa (Fig. 2B). Moreover, the loss of free sulfhydryl was commonly recognized as attributed to disulfide bonds in proteins formed between the free sulfhydryl groups [34]. The results suggested that Laccase might increase the free sulfhydryl oxidation in the production of whole-wheat bread.

### Determination of the Specific Volume of the Breads

**Fig. 3:**
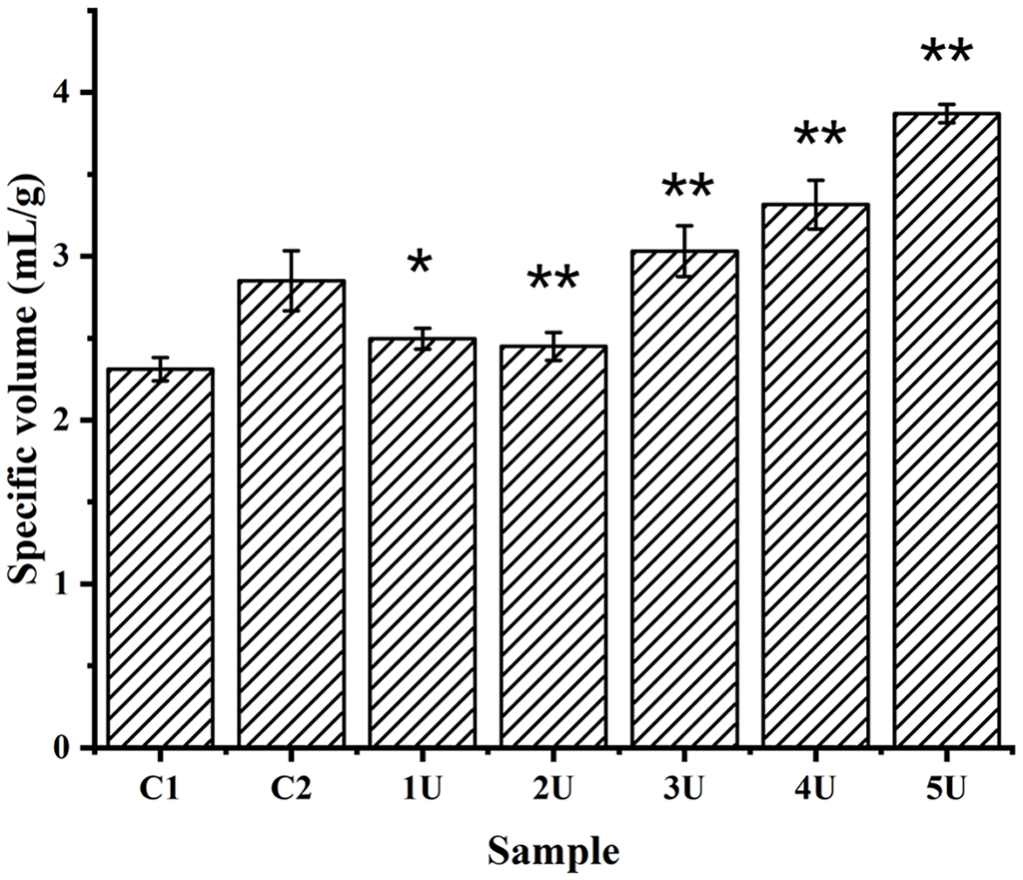
Bread specific volume histogram. C1 control group represents the whole wheat-added sterile water, and C2 control group represents the whole wheat-added phosphate buffer. The 1 U, 2 U, 3 U, 4 U and 5 U represent the experimental groups with LacHa addition of 1 U, 2 U, 3 U, 4 U, and 5 U in each group (*p < 0.05, **p < 0.01, one-way ANOVA, *n* = 3).

The specific volume of whole-wheat bread with LacHa was different from that in the control groups. When the addition of LacHa is greater than 4U, the average specific volume of the bread increases significantly. The specific volume of the whole-wheat bread reached the maximum at approximately 5 U with the addition of LacHa, compared with the bread without laccase and phosphate buffer (2.31–3.87 ml/g), showing an increase of 1.56 ml/g (Fig. 3). In addition, the specific volume of the control group with phosphate buffer (C2) was significantly higher than that of the control group without phosphate buffer (C1), which might be related to the stability of the bread structure with phosphate buffer solution. These results showed that the addition of LacHa to whole-wheat bread might increase the strength and volume of dough significantly (Fig. 3).

In verifying the accuracy of this test method, the length, width, and height of the bread to be tested were measured three times for each sample, and the final results were averaged and compared with the data of the millet displacement volume method. The data were close to the actual volume of the bread, and the method is considered highly accurate and reliable (Fig. S1).

### Determination of Bread Texture Parameters

**Fig. 4:**
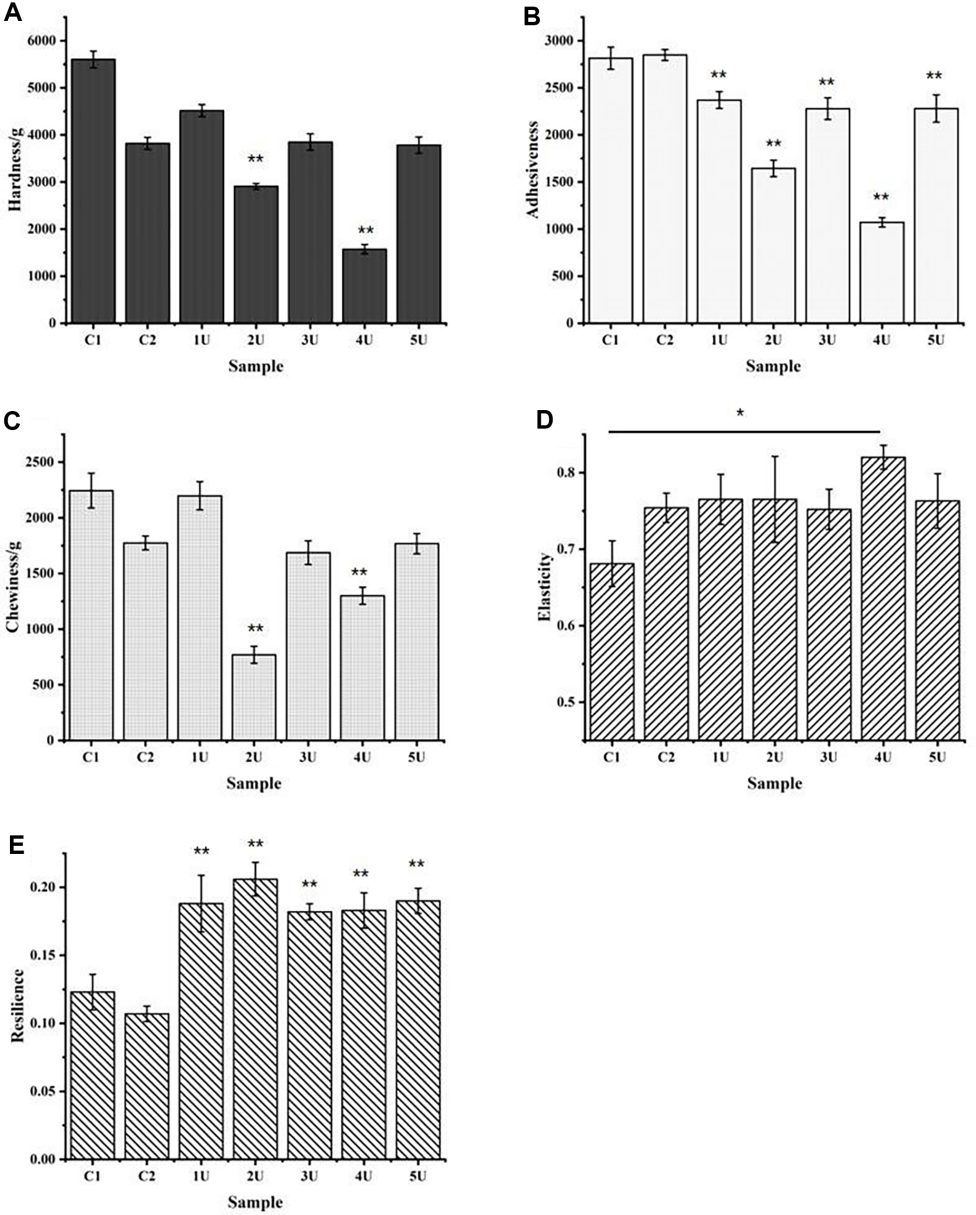
Histogram of whole-wheat bread texture characteristics. Figs. 4a-4e show the hardness, adhesiveness, chewiness, elasticity and resilience of whole-wheat bread with the increase in the amount of LacHa added, respectively. C1 control group represents the whole wheat-added sterile water, and C2 control group represents the whole wheat-added phosphate buffer. The 1 U, 2 U, 3 U, 4 U and 5 U represent the experimental groups with LacHa addition of 1 U, 2 U, 3 U, 4 U, and 5 U in each group, respectively (*p < 0.05, **p < 0.01, one-way ANOVA, *n* = 3).

The quality of whole-wheat bread is not directly proportional to the level of LacHa addition. The hardness, adhesiveness, and chewiness of the bread decreased significantly to 1573.835 g, 1071.463, and 1300.264 g, respectively, when the amount of LacHa added was 4 U. In addition, the resilience at the 2 U addition level reached the maximum, which was significantly different from the two control groups (Figs. 4A-4C). However, when the level of enzyme increased to 5U, the parameters, including hardness, adhesiveness, and chewiness, increased correspondingly. Therefore, the quality of whole-wheat bread was best when the amount of LacHa added was 4 U, and the hardness, adhesiveness, and chewiness were 1 to 3 times lower than those of the two control groups (Figs. 4A-4C). For the two control groups, the hardness and chewiness of whole-wheat bread containing phosphate buffer were lower than that of whole-wheat bread without phosphate buffer (Figs. 4A-4C). The hardness, adhesiveness, and chewiness of whole-wheat bread are negatively correlated with the quality of whole-wheat bread, but the elasticity and resilience are significantly and positively correlated with bread quality. Adding 4 U LacHa, the elasticity of whole-wheat bread reached the maximum of 0.820, while the resilience of the bread reached the maximum of 0.206 with 2 U LacHa. However, by increasing the amount of enzyme added, the resilience slightly decreases (Figs. 4D and 4E).

### Analysis of Bread Storage Duration

**Fig. 5:**
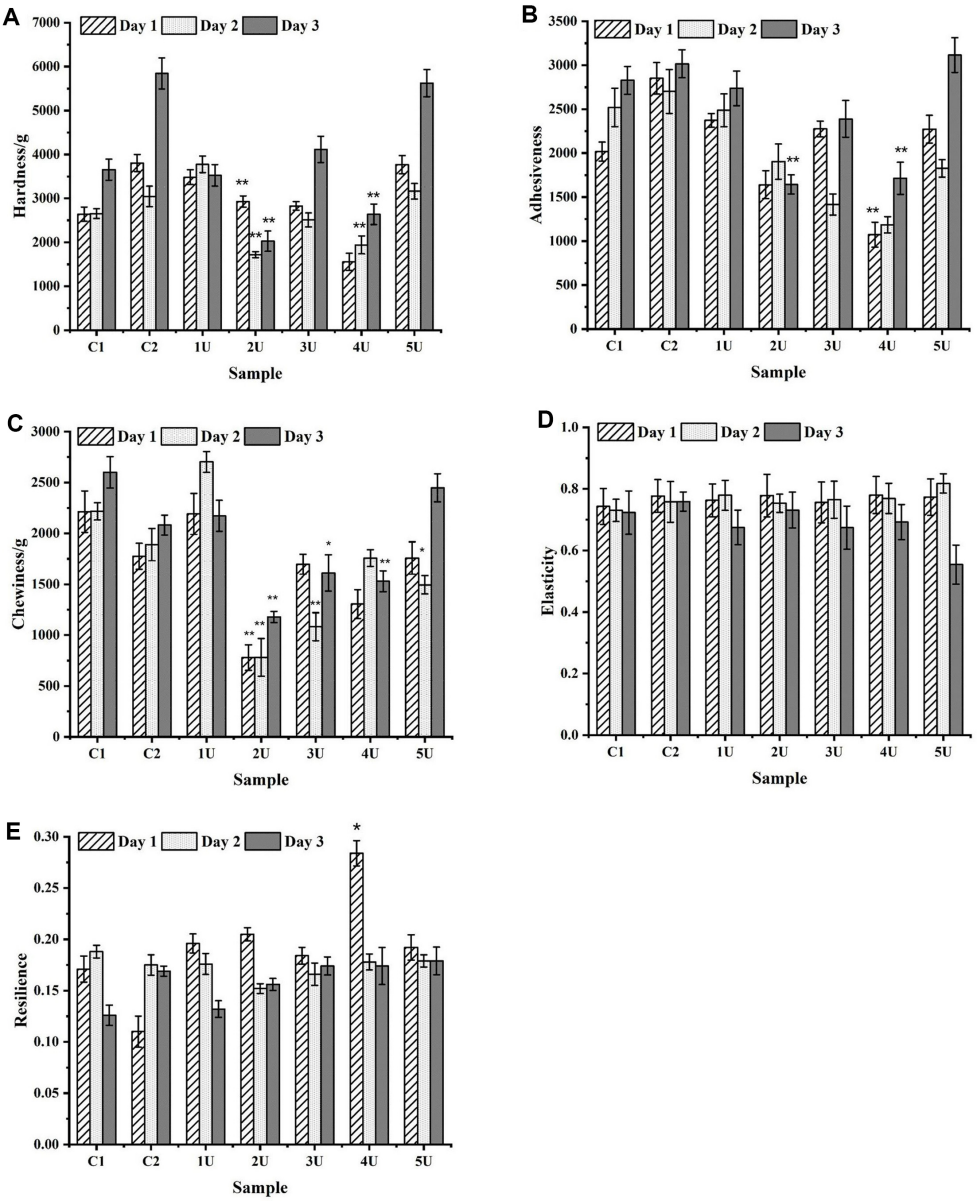
Variation of whole-wheat bread texture characteristics of each group with storage time. Figs. 5A-5E show the changes of whole-wheat bread hardness, adhesiveness, chewiness, elasticity, and resilience with storage time, respectively. C1 control group represents the whole wheat-added sterile water, and C2 control group represents the whole wheat-added phosphate buffer. The 1 U, 2 U, 3 U, 4 U, and 5 U represent the experimental groups with LacHa addition of 1 U, 2 U, 3 U, 4 U, and 5 U in each group, respectively (*p < 0.05, **p < 0.01, one-way ANOVA, *n* = 3).

The minimum values of the hardness, adhesiveness, and chewiness of whole-wheat bread are reached on the first day or the second day, and show an upward trend over time. The elasticity and resilience also show a downward trend. On the third day of storage, the hardness, adhesiveness and chewiness of whole-wheat bread at 4 U LacHa increased to 2,631.54, 1,710.76, and 1,531.63 g, respectively, which represented significantly lower values than those of the two control groups (Figs. 5A-5C). Meanwhile, the resilience was reduced to 0.174, which was still greater than the values measured on the first day for the two control groups (Figs. 5D and 5E). Similarly, on the third day of storage, the chewiness, elasticity and resilience of C2 group containing phosphate buffer were also better than that of C1 without phosphate (Figs. 5A-5E). These results indicated that the addition of LacHa could slow the aging rate of bread.

## Discussion

In this study, two control groups were selected, namely, non-added components in whole-wheat bread and whole-wheat bread with phosphate buffer, to eliminate the possible impact of phosphate buffer in the laccase reaction generation system on the quality of the breads. The results showed that the bread in the control group with phosphate-buffered solution was superior to the control group without phosphate-buffered solution in terms of the specific volume, hardness, chewiness, and elasticity (Figs. 3 and 4A-4E-4E). This result might be due to the hydrophilicity of the phosphate group. The water retention of bread was greatly improved after adding phosphate buffer, which further improved the other qualities of bread. For example, modified starch is more hydrophilic after phosphorylation [35]. Compared with the whole-wheat bread without phosphate, the structure of the whole-wheat bread with phosphate was more compact, and the extraction rate of gluten protein was lower, which indicated that the phosphate-buffered solution can improve bread quality, as shown by SEM, gluten protein extraction, and the free sulfhydryl group content analysis (Figs. 1 and 2).

Given the unique properties of whole-wheat bread, the bran mixed in with the gluten protein will shear the gluten protein in the dough, destroy the formed gluten protein, and hinder its formation of a three-dimensional network structure [8, 9]. In this study, when a certain amount of LacHa, is added, the specific volume and most texture properties of whole-wheat bread were significantly better than those of the two control groups without LacHa (Figs. 1A, 1b, 2, and 4A-4E). Based on previous reports, laccase can promote the cross-linking polymerization of gluten protein in whole-wheat dough, which makes baked whole-wheat bread fluffy, while also increasing bread volume and specific volume [36]. For example, when laccase was added to oat bread, the cross-linking degree of dough increased with the increase of laccase concentration, and the volume of oat bread was larger than that of the control group [16, 37]. Specific volume and hardness, the primary parameters influencing consumer acceptance of bread, were significantly affected by enzyme addition [38]. In this study, the hardness of whole-wheat bread is negatively correlated with the specific volume of bread: the larger the volume of bread, the lower the degree of hardness (Table S1).

Water in dough is present in three forms: tightly bound, less tightly bound, and free water. Tightly bound and less tightly bound water is bound by some hydrophilic groups and its movement is restricted, while most of the water in food is in a free state [39, 49]. In dough, water interacts with gluten and starch to form a “bicontinuous network” – a continuous water-containing gluten phase that is interpenetrated by the continuous free water starch mixture phase [39]. Laccase can accelerate the enzymatic reaction of whole-wheat dough, which cross-links with the sulfhydryl group in gluten protein, and other macromolecular substances in the dough combined with water; thus, part of the free water in the dough structure is fixed [23]. Dough with good water retention not only retains water, but also promotes the combination of components and water in the dough; thus, the nutrients in the dough are not easily lost. Ferulic acid in whole-wheat flour will interact with gluten, react with gluten protein, and destroy the structure of dough [23]. Cura’s research suggests that adding laccase to flour can oxidize ferulic acid and reduce its destructive effect on dough [2]. In addition, the cross-linking reaction between laccase and ferulic acid produces gel to retain water molecules, which can improve the water retention performance of dough [2].

Moreover, laccase induces cross-linking of proteins during baking, which oxidizes sulfhydryl groups in dough into disulfide bonds and improves the elasticity and hardness of the dough [16, 40-42]. This finding was also reflected in the texture analysis of whole-wheat bread in this study. The harder whole-wheat bread is, the less elasticity it has, and the harder it tastes. The greater the chewiness, the harder the bread is to chew, and the lower the quality of the bread. The less sticky the bread is, the softer it is. Moreover, as the storage time increased (3 days), the quality of whole-wheat bread, including the control and experimental groups, all decreased. However, with LacHa added, the properties of whole-wheat bread changed more slowly than those of the control. The reason was that the addition of laccase reduced the hardness of bread and increased the elasticity (Figs. 4A-4E). Studies have shown that only high doses of laccase or a combination of laccase and xylanase can soften bread quality, possibly due to the excessive oxidation of polyphenols or arabinoxylans [41]. In this study, only a single and low amount of LacHa was used to improve the quality and shelf life of whole-wheat bread.

Along with SEM observation, the extractable rate of gluten protein and the free sulfhydryl group content were selected to analyze the structure of whole-wheat dough in this experiment. The SEM results revealed that whole-wheat bread with LacHa showed tightly combined gluten and starch granules, forming a smooth and flat structure (Fig. 1). Compared with the two control groups, the extractable rate of gluten protein in whole-wheat bread was also the lowest when LacHa reached 4 U, and the free sulfhydryl content of the doughs decreased significantly with the addition of LacHa (Fig. 2). Based on previous reports, laccase promoted the degree of protein cross-linking in black highland barley noodles and gluten-free amadumbe flour [13, 23, 43]. Labat *et al*. also suggested that the laccase might participate in a redox reaction involving free sulfhydryl groups to promote protein cross-linking and catalyze the formation of covalent bonds [35]. Therefore, LacHa was considered to enhance the interaction among proteins in whole-wheat dough.

In this study, the obtained LacHa differed from the laccases of the previous reports. At present, most of the laccases used in food baking come from fungi and often have a higher specific enzyme activity, such as *Aspergillus oryzae*, *Trametes versicolor*, *Trametes maxima* CU1, and *Trametes hirsute* [13, 15, 51] (Table S2). Although the catalytic reaction is similar, great differences in the properties of fungal laccase and bacterial laccase were observed. Bacterial laccases exhibited rather low redox potential as compared with fungal laccases [44]. However, the long production cycle, poor thermostability, and low tolerance to the alkaline conditions hindered the practical application of fungal laccases [45]. Recently, bacterial laccases have shown advantageous characteristics, including good stability under high temperatures and alkaline conditions [46, 47]. In addition, using a redox mediator, bacterial laccases could degrade the recalcitrant substrates with higher redox potential than that of fungal laccases [48, 49]. Previous studies showed that LacHa had many excellent characteristics, for example, the potential advantages of activity and stability under high temperatures and alkaline pH conditions, as well as great chloride tolerance. Moreover, LacHa could efficiently oxidize representative indigo carmine and azo dyes used in textile industries without any mediators (data not available). Studies have also shown that when laccase is mixed with other substances (*e.g.*, tyrosinase and laccase, arabinoxylans and laccase, or laccase and xylanase), it can further improve the properties of dough and strengthen the coaching role of the inner protein of dough (Table S2). In this study, a separate and lower amount of LacHa (4 U/100 g flour) also improved the quality of whole-wheat bread, and provided a good theoretical and practical foundation for the application of laccase in the food processing industry.

## Conclusion

Pure LacHa was used to study the effects of quality parameters of the whole-wheat bread, including specific volume, texture analysis, and storage. The results showed that with the addition of a certain amount of LacHa, the specific volume of whole-wheat bread significantly increased compared with the control groups; the resilience of bread treated with LacHa was significantly increased compared with the control groups, whereas the hardness, adhesiveness, and chewiness were significantly reduced, which made the bread taste better and denser. The optimum addition of LacHa for whole-wheat bread was approximately 4 U, and the properties of bread were optimal under this condition. After 3 days of storage, the bread quality with LacHa was better than that of the control bread, and the bread staling rate was reduced; so the addition of LacHa prolongs the shelf life of bread. Based on SEM, the extractable rate of gluten protein and free sulfhydryl group content analyses, LacHa could improve the quality parameters of whole-wheat bread by improving gluten cross-linking. Therefore, laccase can effectively optimize the properties of whole-wheat bread and greatly improve its taste.

## Supplemental Materials

Supplementary data for this paper are available on-line only at http://jmb.or.kr.
