# Supplementary material for: Effect of a Bacterial Laccase on the Quality and Micro-Structure of Whole Wheat Bread
Source: J Microbiol Biotechnol. 2023 Jul 28;33(12):1671–80. doi: 10.4014/jmb.2305.05008 (PMC10772560; doi:10.4014/jmb.2305.05008)
Supplement: Supplementary file 1 [file jmb-33-12-1671-supple.pdf]

J. Microbiol. Biotechnol.
https://doi.org/10.4014/jmb.2305.05008

Supplementary Figure and Table

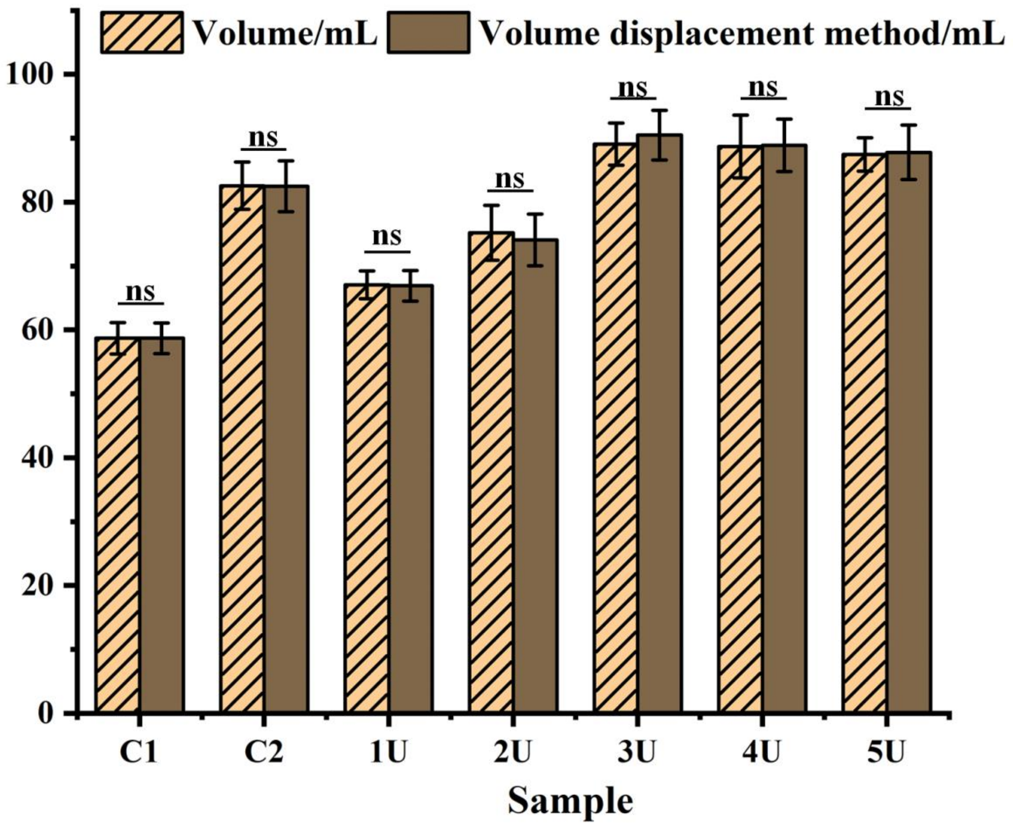

Figure. S1. Comparison of volumetric and foxtail millet volumetric permutation data. C1 in the figure represents the whole wheat + sterile water control group. C2 in the figure represents the whole wheat + phosphate buffer control group. The 1U, 2U, 3U, 4U and 5U in the figure represent the experimental groups with LacHa addition of 1U, 2U, 3U, 4U and 5U in each group, respectively.

Table S1. Correlations between hardness and volume.

		Volume	Hardness
Volume	Pearson Correlation	1	-.764*
	Sig. (2-tailed)		.046
	N	7	7
Hardness	Pearson Correlation	-.764*	1
	Sig. (2-tailed)	.046	
	N	7	7

*. Correlation is significant at the 0.05 level (2-tailed).

Table S2. Summary table of laccase application in food processing field.

Source strain	Enzyme activity	Optimum pH	Molecular weight
Trametes hirsute[1]	149.9 U/mg	4.5	/
Trametes versicolor[2]	0.5 U/g	5.5	45
Aspergillus oryzae [3]	800 U/g	4.5	56
Myceliophthora thermophila [4]	3.13 U/mg	4.5	140
Aspergillus oryzae[5]	30 U/mg	5.0	58
Pycnoorus sanguineus[6]	1642 U/ml	3.0	37

References

1. Selinheimo E, Autio K, Kruus K, Buchert J. 2007. Elucidating the Mechanism of Laccase and Tyrosinase in Wheat Bread Making. *J. Agric. Food Chem.* **55**: 6357-6365.
2. Alizadeh-Bahaabadi G, Lakzadeh L, Forootanfar H, Akhavan HR. 2022. Optimization of gluten-free bread production with low aflatoxin level based on quinoa flour containing xanthan gum and laccase enzyme. *Int. J. Biol. Macromol*. **200**: 61-76.
3. Serventi L, Skibsted LH, Kidmose U. 2016. Individual and combined effects of water addition with xylanases and laccase on the loaf quality of composite wheat–cassava bread. *Eur. Food Res. Technol*. **242**: 1663–1672.
4. Renzetti S, Courtin CM, Delcour JA, Arendt EK. 2010. Oxidative and proteolytic enzyme preparations as promising improvers for oat bread formulations: Rheological, biochemical and microstructural background. *Food*

Chem. **119**: 1465-1473.

5. Fan JX, Guo XN, Zhu KX. 2022. Impact of laccase-induced protein cross-linking on the in vitro starch digestion of black highland barley noodles. *Food Hydrocolloids* **124**: 107298.
6. Ayala-Soto F, Serna-Saldívar S, Welti-Chanes J. 2017. Effect of arabinoxylans and laccase on batter rheology and quality of yeast-leavened gluten-free breads. *J. Cereal Sci*. **73**: 10-17.
